# Rao and Wald Tests for Adaptive Detection in Partially Homogeneous Environment with a Diversely Polarized Antenna

**DOI:** 10.1155/2013/369103

**Published:** 2013-09-18

**Authors:** Chaozhu Zhang, Jing Zhang, Chengyuan Liu

**Affiliations:** College of Information and Communications Engineering, Harbin Engineering University, Harbin, Heilongjiang 150001, China

## Abstract

This study considers Rao test and Wald test for adaptive detection based on a diversely polarized antenna (DPA) in partially homogeneous environment. The theoretical expressions for the probability of false alarm and detection are derived, and constant false alarm rate (CFAR) behaviour is remarked on. Furthermore, the monotonicities of detection probability of the two detectors are proved, and a polarization optimization detection algorithm to enhance the detection performance is proposed. The numerical simulations are conducted to attest to the validity of the above theoretical analysis and illustrate the improvement in the detection performance of the proposed optimization algorithm.

## 1. Introduction

Detecting a signal of interest in the presence of noise is often encountered in radar/sonar signal processing. In an ideal situation, the noise in the training data is assumed to share the same covariance matrix as that in the test data. This situation is often referred to as a homogeneous environment. In this case, many classic algorithms, such as the generalized likelihood ratio test (GLRT) detector [1] and adaptive matched filter (AMF) detector [2], are widely used. A prominent feature of the two detectors is constant false alarm rate (CFAR). The GLRT detector is obtained through replacing all the unknown parameters with their maximum likelihood (ML) estimates under each hypothesis within one step [1]. We refer to this procedure as a one-step design procedure. On the opposite, the AMF detector is derived with an ad hoc two-step design procedure [2]. In other words, this design procedure is the first to assume that the noise covariance matrix is known, and to obtain the GLRT test by maximizing over other unknown parameters. The ML estimate of the noise covariance matrix based on the training data alone is then substituted into this test. In the following, the GLRT and AMF detectors are referred to as the one-step and two-step GLRT-based detectors, respectively.

However, nonhomogeneous environments may be encountered in many practical applications [3]. In airborne radars, for instance, the ground clutter in the test and training data is generated by reflections from different portions of the ground. The power level fluctuations of the ground clutter might arise due to variations in terrain [4]. Several models have been proposed for the nonhomogeneous environments [5]. One of these models is the partially homogeneous model, where the noise covariance matrix has the same structure in the test and training data samples but may differ by a scaling factor. This partially homogeneous model is also used in a wireless communication system with fades over multiple sources of interference [6]. The detection problems in the partially homogeneous environments have recently attracted much attention [6–17]. Many detection algorithms, such as matched subspace detector (MSD) [12] and adaptive subspace detector (ASD) [13], are proposed to deal with detection problems in the partially homogeneous environment. Notice that the ASDs contain the common one-step and two-step GLR-based detectors for the case in which the scaling factor of the test data may deviate from that of the training data [3]. Additionally, the CFAR ASD has been proved to be a uniformly most powerful invariant test [6, 17]. Interestingly, the CFAR ASD exhibits great robustness to the scaling of the test data, whereas Kelly's GLR detector [1] and Robey's AMF [2] are sensitive to the scaling factor and even cease to be CFAR [6]. Reference [16] showed that the CFAR ASD is GLRT when the test measurement is not constrained to have the same noise level as the training data.

Moreover, the GLRT has been the most commonly employed one in signal processing. Nevertheless, since there is no particular a priori reason to exploit the GLRT rather than the others, during the last three decades, Rao test [18] and Wald test [19] applied to practical signal processing detection problems have started to appear in open literature. For instance, in [20], an adaptive detector based on Rao criterion is devised to discriminate the presence of a deterministic signal, with unknown amplitude, in Gaussian noise with unknown but structured (AR parameterized) spectra. In [21, 22], Rao and Wald tests are, respectively, derived to detect a signal with unknown amplitude in homogeneous Gaussian disturbance with unknown covariance matrix. In [23], Rao test has been applied to the problem of radar space-time adaptive processing (STAP), while in [24, 25], Rao and Wald tests are devised with reference to adaptive detection of distributed targets in non-Gaussian clutter. In [26–28], Rao and Wald tests have been applied to multiple-input multiple-output radar detection in compound-Gaussian clutter. Finally, in [29, 30], the coincidence and statistical equivalence of the GLRT, Rao test, and Wald test are proved.

As is well known, a diversely polarized antenna (DPA) has some inherent advantages over a scalar sensor, since it can handle signals based on their polarization characteristics. The tripole antenna in this paper is a common diversely polarized antenna, and it consists of three mutually perpendicular short dipoles—all centered at the same location, and it has as its output the three components of the impinging electric filed [31].

Generally, the performance of a radar system is associated with many characteristics of the transmitted signals, such as polarization [32]. Therefore, the system performance (for example, detection capability) can be improved by optimally selecting the transmitted signals. In [33], the authors have developed a polarimetric detector based only on several primary data vectors and shown that this test statistic has the standard *F*-distribution. Hence, the detection performance of the polarimetric detector can be improved by optimally choosing the polarization of the transmitted pulses to maximize the noncentrality parameter. Based on a diversely polarized antenna, [34, 35] addressed the problems of adaptive detection and performance enhancement by optimally selecting the polarization of the transmitted pulses of the DPA in homogeneous and partially homogeneous environments. Hence, the detection performance of the polarimetric detector can be improved by optimally choosing the polarization of the transmitted pulses to maximize the noncentrality parameter.

In this paper, we study Rao test and Wald test for adaptive detection based on a DPA in partially homogeneous environment. Our main contribution is to derive the expressions for the probabilities of false alarm and detection of Rao test and Wald test with unknown noise covariance matrix structure based on a DPA, in the partially homogeneous environment. It is found from these expressions derived that Rao test and Wald test have the CFAR property. The other contribution of this paper is that the monotonicities of detection probability of the two detectors are proved and a polarization optimization detection algorithm to enhance the detection performance is proposed. The improvement in the detection performance of the DPA is achieved by using the proposed algorithm to optimally select the polarization of the transmitted pulses.

The rest of this paper is organised as follows. The statement of the problem and the description of the signal and noise models are given in Section 2. The theoretical expressions for the probability of false alarm and detection of Rao and Wald detectors are derived in Section 3. The monotonicities of detection probability of the two detectors are proved in Section 4. Polarization optimization detection algorithm is proposed in Section 5. The simulation results of the two detectors as well as the GLRT are displayed in Section 6. Lastly, conclusions are given in Section 7.

## 2. Signal Model

In this section, we consider a detection problem in partially homogeneous environments. The received *Q*-dimensional complex vector **x**, commonly called primary data or test data, is constrained to be of the form

(1) $$\begin{array}{c} x=\Sigma s+n, \end{array}$$

where Σ is a known *Q* × *q* dimension signal subspace matrix representing the system response associated with the characteristics of the transmitted signals (e.g., polarization), and suppose that *Q* > *q* and rank⁡(Σ) = *q*; **s** is a *q*-dimensional deterministic but unknown complex vector accounting for the target reflectivity and the channel propagation effects; **n** is a noise data vector and is assumed to have a complex circular Gaussian distribution with zero mean and covariance matrix *μ* **R**, that is, **n** ~ CN(0, *μ* **R**), where **R** is an unknown, positive definite noise covariance matrix structure and *μ* is an unknown scaling of the noise in the test data. Notice that the scaling factor accounts for the noise power mismatch between the primary and secondary data. The arbitrary scaling between the primary and secondary data is important in some realistic scenarios [3].

Suppose that *K*  (*K* > *Q*) secondary data samples free of the target signal, that is {**y**_*k*_, *k* = 1,…, *K* | **y**_*k*_ ~ CN(0, **R**)}, are available. The problem of detection is to decide whether the target signal is present or not in the range cell under test. This problem can be posed in terms of a binary hypotheses test. We let the null hypothesis (*H*_0_) be that no target signal is present and let the alternative hypothesis (*H*_1_) be that the data contains target signal. Hence, the detection problem is to decide between the null hypothesis and the alternative one and can be stated as a parameter test:

(2) $$\begin{array}{c} H_{0}:\{\begin{array}{cc} x\sim \text{CN}\left(0,\mu R\right), & \\ y_{k}\sim \text{CN}\left(0,R\right), & k=1,\ldots ,K, \end{array}\\ H_{1}:\{\begin{array}{cc} x\sim \text{CN}\left(\Sigma s,\mu R\right), & \\ y_{k}\sim \text{CN}\left(0,R\right), & k=1,\ldots ,K. \end{array} \end{array}$$

## 3. Adaptive detectors

Now, a brief introduction about ASD is explained in order to study Rao test and Wald test performances.

In practice, a prior knowledge on the covariance matrix structure is usually unknown. According to [35], the detector used to handle the detection problem with unknown **R**, which is referred to as ASD, is

(3) Ψ=xHR^−1Σ(ΣHR^−1Σ)−1ΣHR^−1xxHR^−1x≶H1H0gASD,

where *g*_ASD_ ∈ (0,1) is the detection threshold and R^=∑k=1KykykH. The superscript “*H*” denotes complex conjugation transpose.

The false alarm probability function of ASD can be written as

(4) $$\begin{array}{c} F\left(g_{\text{ASD}}\right)=P_{\text{FA}}^{\text{ASD}}\left\{\Psi >g_{\text{ASD}}{|}_{H_{0}}\right\}=\overset{1}{\underset{0}{\int }}P_{\text{FA}\;|\;\rho }^{\text{ASD}}f_{\rho }\left(\rho \right)d\rho , \end{array}$$

where *ρ* denotes a loss factor whose distribution is

(5) $$\begin{array}{c} f_{\rho }\left(\rho \right)=\frac{K!\rho^{K-Q+q}{\left(1-\rho \right)}^{Q-q-1}}{\left(Q-q-1\right)!\left(K-Q+q\right)!},\;0<\rho <1,\\ P_{\text{FA}\;|\;\rho }^{\text{ASD}}={\left(\frac{1-g_{\text{ASD}}}{1-g_{\text{ASD}}\rho }\right)}^{K-Q+1}{\overset{q}{\underset{j=1}{\sum }}C_{K-Q+q-j}^{q-j}\left[\frac{g_{\text{ASD}}(1-\rho )}{1-g_{\text{ASD}}\rho }\right]}^{q-j}. \end{array}$$

Furthermore, the probability detection function of ASD is [3]

(6) $$\begin{array}{c} Z\left(g_{\text{ASD}}\right)=P_{D}^{\text{ASD}}\left\{\Phi >g_{\text{ASD}}{|}_{H_{1}}\right\}=\overset{1}{\underset{0}{\int }}P_{D\;|\;\rho }^{\text{ASD}}f_{\rho }\left(\rho \right)d\rho , \end{array}$$

where the probability of detection conditioned on *ρ* is

(7) PD ∣ ρASD=1−[gASD(1−ρ)1−gASDρ]q−1(1−gASD1−gASDρ)K−Q+1 ×∑j=1K−Q+1CK−Q+qq+j−1[gASD(1−ρ)1−gASD]j ×exp⁡[−Γρ(1−gASD)1−gASDρ]∑m=0j−11m![Γρ(1−gASD)1−gASDρ]m,Γ=sH[ΣH(μR)−1Σ]s.

Reference [29] proved that Rao test, Wald test, and GLRT [16] coincide in the presence of partially homogeneous complex circular Gaussian disturbance with unknown covariance matrix and Rao test which can be written as

(8) $$\begin{array}{c} \Phi =\frac{Q\left(K+1-Q\right)\Psi }{\left(K+1\right)\left(K+1+Q\Psi \right)}\overset{H_{0}}{\underset{H_{1}}{≶}}g_{R,1} \end{array}$$

(9) $$\begin{array}{c} \Leftrightarrow \Psi \overset{H_{0}}{\underset{H_{1}}{≶}}g_{R,2}=\frac{g_{R,1}{\left(K+1\right)}^{2}}{Q\left[K+1-Q-\left(K+1\right)g_{R,1}\right]}, \end{array}$$

where *g*_*R*,1_ ∈ (0, *Q*(*K* + 1 − *Q*)/(*K* + 1)(*K* + 1 + *Q*)) denotes the appropriate modification of thethreshold in [29]. Let *α* = (*Q*(*K* + 1 − *Q*) − (*K* + 1)(*Q* + *K* + 1)*g*_*R*,1_)/(*Q*(*K* + 1 − *Q*) − (*K* + 1)(*Q* + *ρK*+  *ρ*)*g*_*R*,1_)  (0 < *α* < 1). The false alarm probability of Rao test can be written as

(10) PFARao{Φ>gR,1|H0} =PFAASD{Ψ>gR,2|H0} =PFAASD{Ψ>gR,1(K+1)2Q[K+1−Q−(K+1)gR,1]|H0} =F(gR,1(K+1)2Q[K+1−Q−(K+1)gR,1]) =∫01PFA|ρRaofρ(ρ)dρ,

where

(11) $$\begin{array}{c} P_{\text{FA}\;|\;\rho }^{\text{Rao}}=\alpha^{K-Q+1}\overset{q}{\underset{j=1}{\sum }}C_{K-Q+q-j}^{q-j}{\left(1-\alpha \right)}^{q-j}. \end{array}$$

The detection probability of Rao test can be written as

(12) PDRao{Φ>gR,1|H1} =PDASD{Ψ>gR,2|H1} =PDASD{Ψ>gR,1(K+1)2Q[K+1−Q−(K+1)gR,1]|H1} =Z(gR,1(K+1)2Q[K+1−Q−(K+1)gR,1]) =∫01PD ∣ ρRaofρ(ρ)dρ,

where

(13) PD ∣ ρRao=1−(1−α)q−1αK−Q+1 ×∑j=1K−Q+1CK−Q+qq+j−1(1−αα)jexp⁡(−Γρα) ×∑m=0j−11m!(Γρα)m.

From [29] we can get Wald test

(14) Ω=QΨ(K+1−Q)(1−Ψ)≶H1H0gW,1

(15) ⇔Ψ≶H1H0gW,2=gW,1(K+1−Q)Q+(K+1−Q)gW,1,

where *g*_*W*,1_ ∈ (0, *∞*) denotes the appropriate modification of the threshold in [29, p. 387, formula (12)]. Let *β* = *Q*/(*Q* + (1 − *ρ*)(*K* + 1 − *Q*)*g*_*W*,1_), 0 < *β* < 1, and the false alarm probability of Wald test can be written as

(16) PFAWald{Ω>gW,1|H0} =PFAASD{Ψ>gW,2|H0} =PFAASD{Ψ>gW,1(K+1−Q)Q+(K+1−Q)gW,1|H0} =F(gW,1(K+1−Q)Q+(K+1−Q)gW,1) =∫01PFA ∣ ρWaldfρ(ρ)dρ,

where

(17) $$\begin{array}{c} P_{\text{FA}\;|\;\rho }^{\text{Wald}}=\beta^{K-Q+1}\overset{q}{\underset{j=1}{\sum }}C_{K-Q+q-j}^{q-j}{\left(1-\beta \right)}^{q-j}. \end{array}$$

The detection probability of Wald test can be written as

(18) PDWald{Ω>gW,1|H1} =PDASD{Ψ>gW,2|H1} =PDASD{Ψ>gW,1(K+1−Q)Q+(K+1−Q)gW,1|H1} =Z(gW,1(K+1−Q)Q+(K+1−Q)gW,1) =∫01PD ∣ ρWaldfρ(ρ)dρ,

where

(19) PD ∣ ρWald=1−(1−β)q−1βK−Q+1 ×∑j=1K−Q+1CK−Q+qq+j−1(1−ββ)jexp⁡(−Γρβ) ×∑m=0j−11m!(Γρβ)m.

From (10) and (16) we can see that Rao test and Wald test have the desirable constant false alarm rate (CFAR) property with respect to both the shared noise covariance matrix structure **R** and the scaling *μ* of the noise in the test data.

## 4. The Monotonicities of Detection Probability

In this section, the monotonicities of detection probability are proved. Firstly, a proposition is introduced, and then a polarization optimization detection algorithm is proposed to enhance the detection performance of the two detectors.

**Proposition 1**

Both *P*_*D*_^*Rao*^ and *P*_*D*_^*Wald*^ are monotonically increasing functions for Γ > 0. (Since **R** ^−1^ is positive definite, we have Γ > 0.)

**Proof**

We transform (14) into an equivalent form as (20) in [35], and then we have 0 < *α* < 1. Using (17), we can obtain

(20) dPDRaodΓ=∫01dPD ∣ ρRaodΓfρ(ρ)dρ=∫01(1−α)q−1αK−Q+1×∑j=1K−Q+1CK−Q+qq+j−1 ×(1−αα)j(ρα)exp⁡(−Γρα)fρ(ρ) ×∑m=0j−1{1m!(Γρα)m−mm!(Γρα)m−1}dρ=∫01(1−α)q−1αK−Q+1×∑j=1K−Q+1CK−Q+qq+j−1 ×(1−αα)j(ρα)exp⁡(−Γρα)fρ(ρ) ×1(j−1)!(Γρα)j−1dρ.

It follows from 0 < *α* < 1 and Γ > 0 that the function of *ρ* in the integral in the right-hand side of (20) is positive for 0 < *ρ* < 1 and is zero for *ρ* = 0 or 1. Thus, the integral of this function over [0,1] is greater than zero; namely, the derivative in (20) is positive.

**Proof**

We transform (8) into an equivalent form as (20) in [35], and then we have *g*_*W*,1_ ∈ (0, *∞*). Using (12), we can obtain

(21) dPDWalddΓ=∫01dPD ∣ ρWalddΓfρ(ρ)dρ=∫01(1−β)q−1βK−Q+1×∑j=1K−Q+1CK−Q+qq+j−1 ×(1−ββ)j(ρβ)exp⁡(−Γρβ)fρ(ρ) ×∑m=0j−1{1m!(Γρβ)m−mm!(Γρβ)m−1}dρ=∫01(1−β)q−1βK−Q+1×∑j=1K−Q+1CK−Q+qq+j−1 ×(1−ββ)j(ρβ)exp⁡(−Γρβ)fρ(ρ) ×1(j−1)!(Γρβ)j−1dρ.

It can be proved in the same way that the derivative in (21) is positive too. The proof is completed.

From the proposition above we can see that the greater the value of Γ  (Γ > 0), the better detection performance. The detection performance of Rao test and Wald test can be enhanced by designing the system response Σ to maximize the parameter Γ. The system response matrix can be parameterized as Σ = Σ(*ε*). The problem of performance enhancement of Rao test and Wald test can be formulated as

(22) $$\begin{array}{c} \;\hat{\varepsilon }=\arg\underset{\varepsilon }{\;\max}\left\{s^{H}\left[\Sigma^{H}\left(\varepsilon \right){\left(\mu R\right)}^{-1}\Sigma \left(\varepsilon \right)\right]s\right\}. \end{array}$$

## 5. Polarization Optimization Detection Algorithm

The matrix **V** is the response of the diversely polarized sensor array [33]. If the array is a tripole antenna, it can be written as

(23) $$\begin{array}{c} V=\left[\begin{array}{cc} -\sin\varphi & -\cos\varphi \sin\psi \\ \cos\varphi & -\sin\varphi \sin\psi \\ 0 & \cos\psi \end{array}\right], \end{array}$$

where *φ* and *ψ* denote the elevation and azimuth angles of the target return with *ϕ* ∈ [0, *π*] and *ψ* ∈ [−*π*, *π*].

The vector **z**_*p*_(*t*) is the *p*th pulse of the narrowband transmitted signal which can be represented by

(24) zp(t)=[z1pz2p]ap(t)=[cos⁡αpsinαp−sinαpcos⁡αp][cos⁡βpjsinβp]ap(t),

where *z*_1*p*_ and *z*_2*p*_ are the signal components on the polarization basis of transmitter, *α*_*p*_ and *β*_*p*_ are the orientation and ellipticity angles of polarization ellipse with *α*_*p*_ ∈ [−*π*/2, *π*/2] and *β*_*p*_ ∈ [−*π*/4, *π*/4], and *a*_*p*_(*t*) (*p* = 1,…, *P*) is the complex envelope of the *p*th transmitted signal pulse and each element of **a**_*p*_ = [*a*_*p*_(*t*_1*p*_),…, *a*_*p*_(*t*_*Mp*_)]^*T*^ (*p* = 1,…, *P*) with *t*_*mp*_  (*m* = 1,…, *M*) denoting the *m*th sampling instant within the *p*th pulse.

The polarization matrix of each diversely polarized pulse (*p* = 1,…, *P*) is given by

(25) $$\begin{array}{c} E_{p}=\left[\begin{array}{ccc} z_{1p} & 0 & z_{2p}\\ 0 & z_{2p} & z_{1p} \end{array}\right]. \end{array}$$

So the system response matrix can be written as

(26) $$\begin{array}{c} \Sigma =\left[\begin{array}{c} a_{1}⊗VE_{1}\\ \vdots \\ a_{P}⊗VE_{P} \end{array}\right], \end{array}$$

and matrix Σ has dimension 3*MP* × 3, where *P* is the number of the transmitted pulses.

The noise covariance matrix is supposed to be **R** = *σ*_*n*_^2^(**I**_*P*_ ⊗ **C**_3*M*×3*M*_), where *σ*_*n*_^2^ is the noise power of each sample, **I**_*P*_ denotes the *P*-dimensional identity matrix, and **C**_*m*×*n*_ is Gaussian shaped with one-lag correlation coefficient *ρ*_*c*_ = 0.9 [35]. That is to say,

(27) $$\begin{array}{c} C_{m\times n}=\left[\begin{array}{cccc} c_{11} & c_{12} & \cdots & c_{1n}\\ \vdots & \vdots & \ddots & \vdots \\ c_{m1} & c_{m2} & \cdots & c_{mn} \end{array}\right], \end{array}$$

where *c*_*ij*_ = 0.9^(*i*−*j*)^2^^, *i* = 1,…, *m*; *j* = 1,…, *n*.

Suppose **R**′ = **I**_*P*_ ⊗ **C**_3*M*×3*M*_ and Γ = (1/*μσ*_*n*_^2^)**s** ^*H*^[Σ^*H*^(**R**′)^−1^Σ]**s**. Note that **R**′ is real symmetric matrix and (**R**′)^−1^ is also real symmetric matrix. So (**R**′)^−1^ can be decomposed into (**R**′)^−1^ = **G** ^*T*^ **G** uniquely, where **G** is 3*MP* × 3*MP* dimension real upper triangular matrix. Therefore, the fitness can be written as

(28) $$\begin{array}{c} \Gamma =\frac{1}{\mu \sigma_{n}^{2}}s^{H}\Sigma^{H}G^{T}G\Sigma s=\frac{1}{\mu \sigma_{n}^{2}}{\left(G\Sigma s\right)}^{H}G\Sigma s. \end{array}$$

Suppose that **H** = **G**Σ**s** and **H** is 3*MP* × 1 dimension complex vector. Then finding the maximum value of Γ is equivalent to finding the maximum modulus value of **H**.

Note that **C**_3*M*×3*M*_ is real symmetric matrix and **C**_3*M*×3*M*_^−1^ is also real symmetric matrix. So it can be decomposed into **C**_3*M*×3*M*_^−1^ = **g** ^*T*^ **g** uniquely, where **g** is 3*M* × 3*M* dimension real upper triangular matrix. Now we have an important discovery: **G** = **I**_*P*_ ⊗ **g**.

**Proof**

From the above analysis, we can get

(29) C3M×3M−1=gTg   ⇔IP⊗C3M×3M−1=IP⊗gTg   ⇔IP−1⊗C3M×3M−1=(IP⊗gT)(IP⊗g)   ⇔(IP⊗C3M×3M)−1=(IP⊗g)T(IP⊗g).

Due to (**I**_*P*_⊗**C**_3*M*×3*M*_)^−1^ = **G** ^*T*^ **G**, we can get

(30) $$\begin{array}{c} G=I_{P}⊗g. \end{array}$$

Then **H** can be written as

(31) H=GΣs=(IP⊗g)Σs=diag⁡[g,…,g][a1⊗VE1⋮aP⊗VEP]s=[ga1⊗VE1s⋮gaP⊗VEPs]=[h1,h2,…,hP]T,

where **h**_*p*_ = **g** **a**_*p*_ ⊗ **V** **E**_*p*_ **s**, *p* = 1,…, *P* is a *P*-dimensional complex vector group, and each one of them is a 3*M* × 1 dimension complex vector.

It is considered in our system that the polarization parameters of different transmitted signal pulses are independent of each other; that is, when *i* ≠ *j*  (*i* = 1,…, *P*; *j* = 1,…, *P*)  (*α*_*i*_, *β*_*i*_) and (*α*_*j*_, *β*_*j*_) are independent of each other. Thus, we can get a conclusion that finding the maximum modulus value of **H** is equivalently decomposed into finding the maximum modulus value of every vector in the complex vector group: **h**_*p*_, *p* = 1,…, *P*.

Now we analyze the complex vector group: **h**_*p*_ = **g** **a**_*p*_ ⊗ **V** **E**_*p*_ **s**, *p* = 1,…, *P*, where real upper triangular matrix **g** is fixed; when transmitted signal pulses and the sampling form are fixed, the complex envelope of the *p*th transmitted signal pulse **a**_*p*_ is fixed; when the target is deterministic, the target reflectivity vector **s** is fixed; in the same pulse interval, we assume that the elevation and azimuth angles of the target fixed, that is, **V**, are fixed. Thus, there are two variable parameters (*α*_*p*_, *β*_*p*_) to be optimized in each vector **h**_*p*_, *p* = 1,…, *P*. Therefore, the proposed algorithm is to optimally choose the parameters (*α*_*p*_, *β*_*p*_) to meet the maximum modulus value of every vector in the complex vector group: **h**_*p*_, *p* = 1,…, *P*.

**Figure 1:**
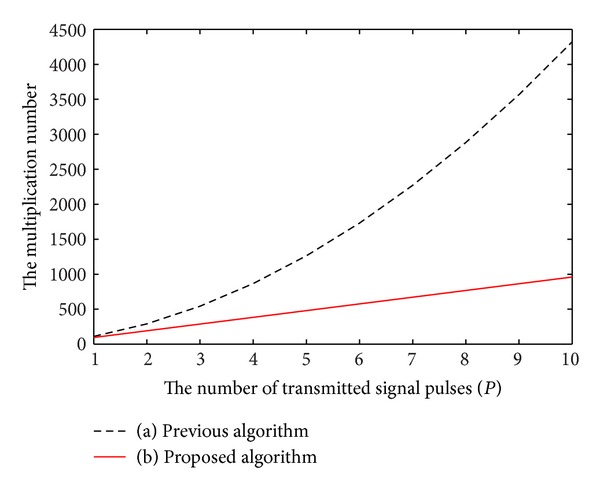
The multiplication numbers of two methods.

The optimization detection algorithm is to find the maximum fitness function value: Γ(*ε*) = **s** ^*H*^[Σ^*H*^(*ε*)(*μ* **R**)^−1^Σ(*ε*)]**s**, and there are *N*_1_ = 9*M* ^2^ *P* ^2^ + 36*MP* + 3 multiplications in the fitness. The proposed algorithm is the equivalently decomposed of previous method [35]. There are *p* fitness functions: **h**_*p*_ = **g** **a**_*p*_ ⊗ **V** **E**_*p*_ **s**, *p* = 1,…, *P*, and they totally have *N*_2_ = 9*M* ^2^ *P* + 30*MP* multiplications. The multiplication number of proposed method is a linear increasing as the parameters increase, while it is a square increasing in the previous method. From Figure 1 we can see that the proposed method is much more efficient than the previous method.

In a special circumstance, *λ*_1_ **a**_1_ = *λ*_2_ **a**_2_ = ⋯ = *λ*_*P*_ **a**_*P*_ = **a**  (*λ*_*k*_ ∈ *R*, *k* = 1,…, *P*), that is, **a**_1_, **a**_2_,…, **a**_*P*_, are linear correlation, for example, rectangular pulses [34]. Thus, we get a conclusion that finding the maximum modulus value of every vector in the complex vector group: **h**_*p*_, *p* = 1,…, *P* is degraded equivalent to finding the maximum modulus value of any vector.

## 6. Experiment Results and Discussions

The experiment results are done by MATLAB program in a PC computer with CPU: inter I3-2100, 3.1 GHz dual-core processor, and 2 GB memory. 

### 6.1. Simulation Results of the Detection Performances

The received data model with a coherent radar in [3] is adopted. We set *Q* = 8, *K* = 48. In order to decrease the computational burden, the probability of false alarm is set to be 0.01.

**Figure 2:**
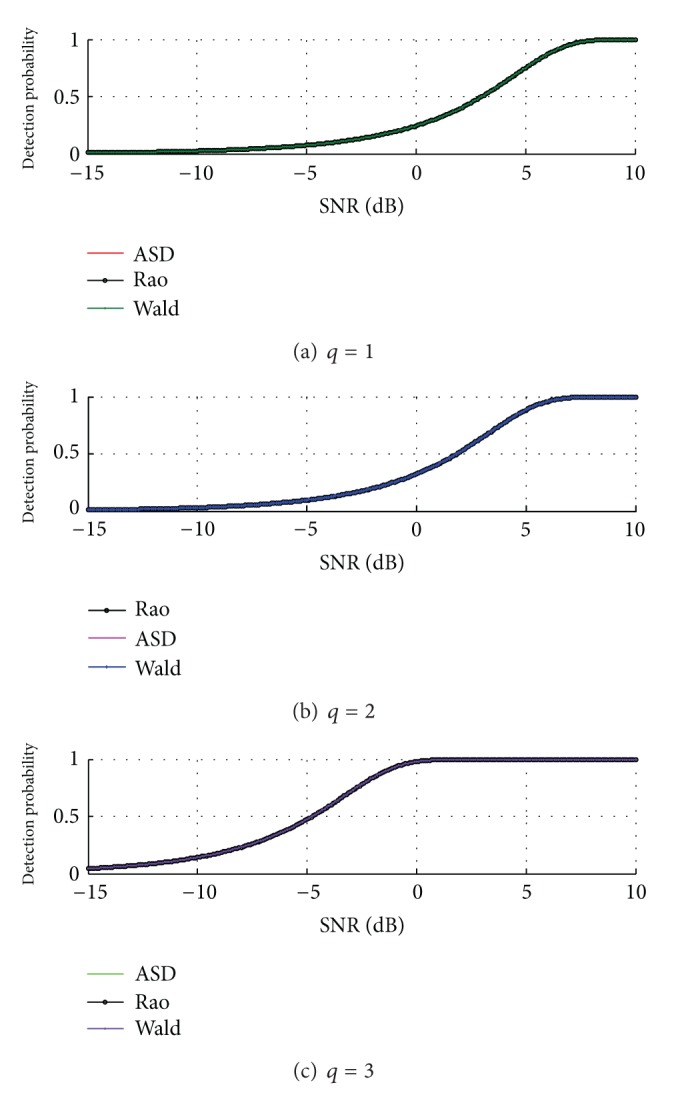
Detection performances of ASD, Rao test and Wald test with different polarimetric channels.

The performances of the ASD, Rao test, and Wald test operating with three polarimetric channels are compared with that of the single and dual channel detectors in Figure 2.

It is shown in Figure 2 that the more the used polarimetric channels, the better the detection performance. In particular, the three-channel detector significantly outperforms the dual-channel detector due to the exploitation of the HV channelwith higher SNR.

**Figure 3:**
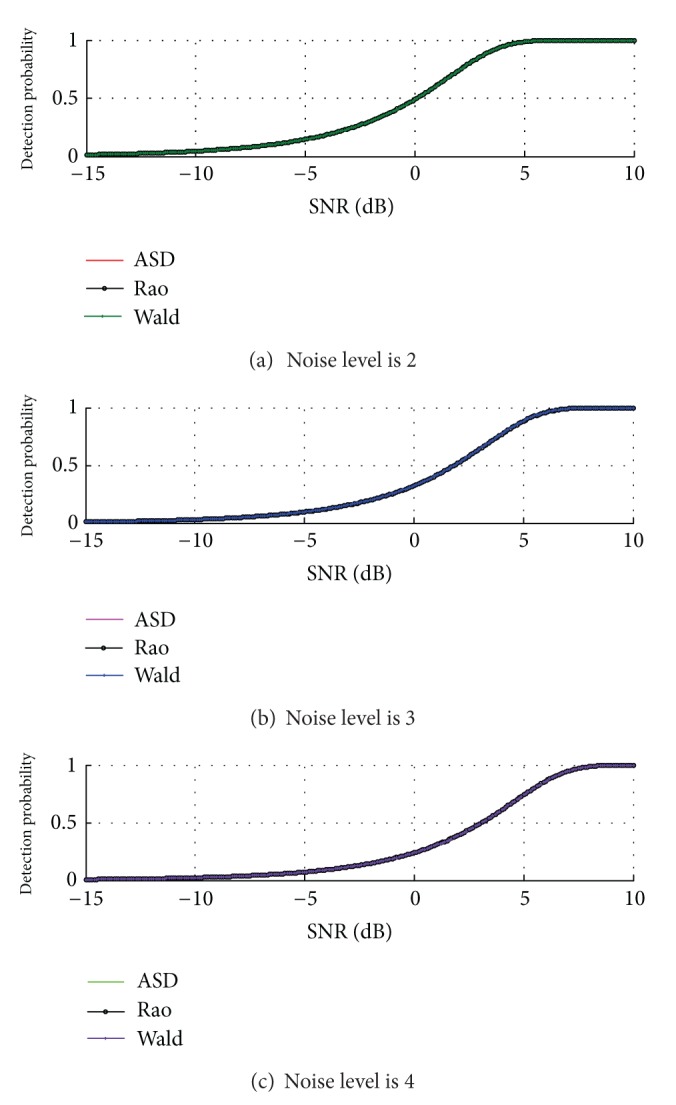
Detection performances of ASD, Rao test and Wald test with *q* = 2 for different noise level *μ* = 2,3, 4.

In Figure 3, the probability of detection of the dual-channel detector as a function of SNR for different values of noise level is plotted. As expected, the increase in the value of *μ* results in a performance loss due to greater noise power received by the radar system.

From Figures 2 and 3 we can see that the detection performance curves of ASD and Rao and Wald tests coincide exactly. For this reason, Figures 2 and 3 are conducted to attest to the coincidence of Rao test, Wald test, and GLRT and illustrate the validity of the expressions for the probabilities of false alarm and detection of Rao test and Wald test with unknown noise covariance matrix structure based on a DPA, in the partially homogeneous environment.

### 6.2. Simulation Results of Detection Performance Optimization Algorithm

In this section, we validated the analytical performance of the algorithms by computer simulations. In the following simulations, we select *P* = 1, 2, 3, 4; *M* = 2; *σ*_*n*_^2^ = 1/3 and *μ* = 3; **s** = [2*i*, −1*i*, 0.5]^*T*^; **a**_1_ = [7 + 8*i*, 8 − 2*i*]^*T*^; **a**_2_ = [5 + 3*i*, 6 − 9*i*]^*T*^; **a**_3_ = [3 + 7*i*, 4 − 4*i*]^*T*^; **a**_4_ = [1 + 5*i*, 2 − 8*i*]^*T*^ in normal circumstance.

The analytical solution can be solved by the proposed method theoretically, but the solution procedure is very complex. Therefore, we use Taguchi optimization algorithm to solve this problem.

**Figure 4:**
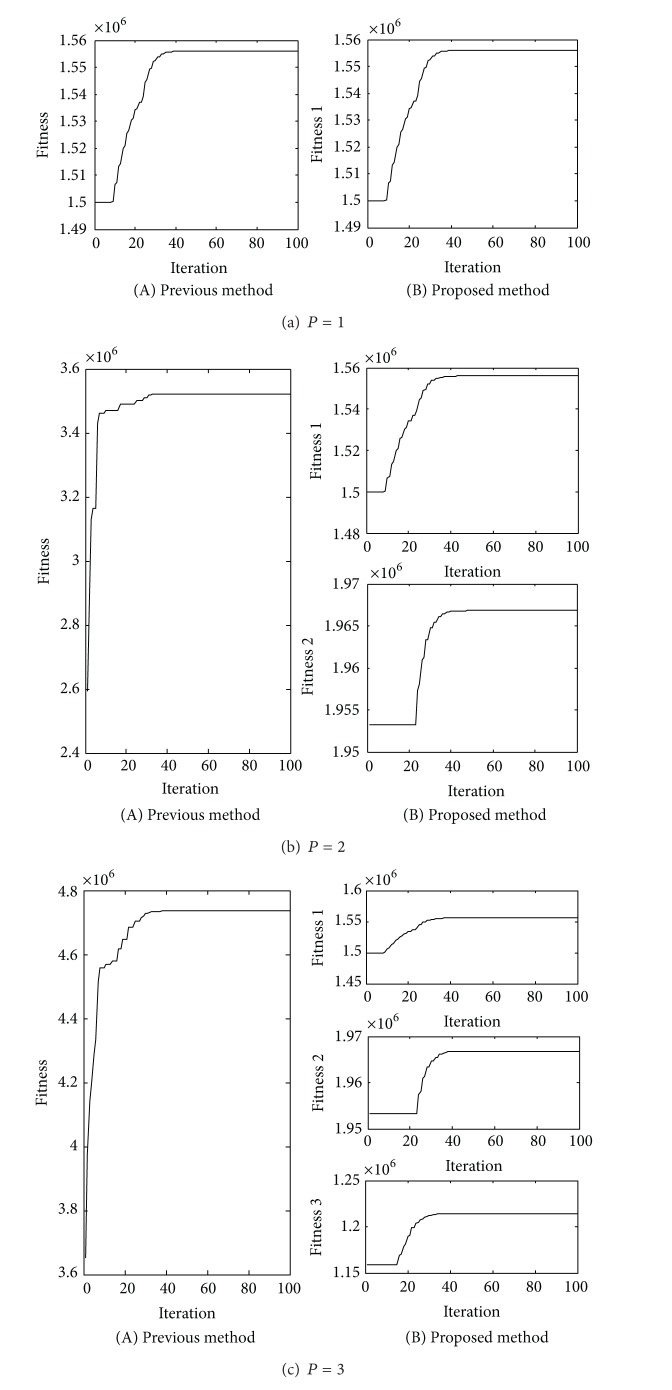

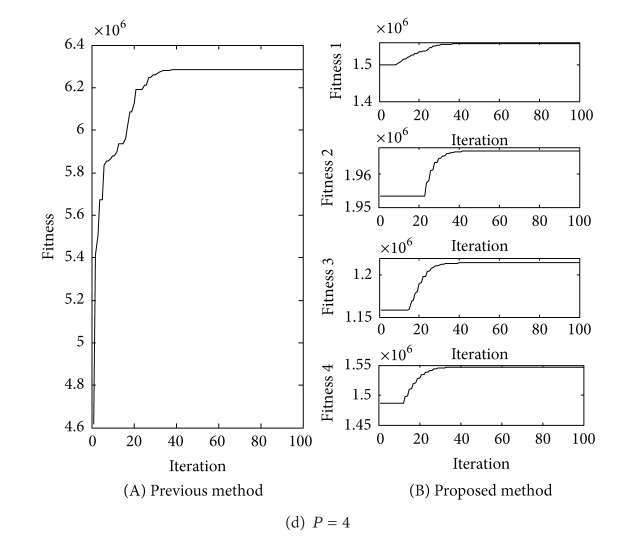
The fitness curves of two methods.

As shown in Figure 4 and Table 1, the sum of the maximum fitness function values in the proposed method is the same as the maximum fitness function value in the previous method. And from Table 2 we can see that the two methods get the same optimal polarization parameters. There is no doubt that the numerical simulations are conducted to attest to the validity of the above theoretical equivalence relation. 

From Table 3 we can see that the proposed method costs less time than the previous method. The numerical simulations confirm the truth that the multiplication number of proposed method is a linear increasing as the parameters increase, while it is a square increasing in the previous method.

From the above theoretical analysis and simulation experiments we can get a conclusion that the proposed method can get the same detection performance as previous method, but it is more efficient than previous method.

**Figure 5:**
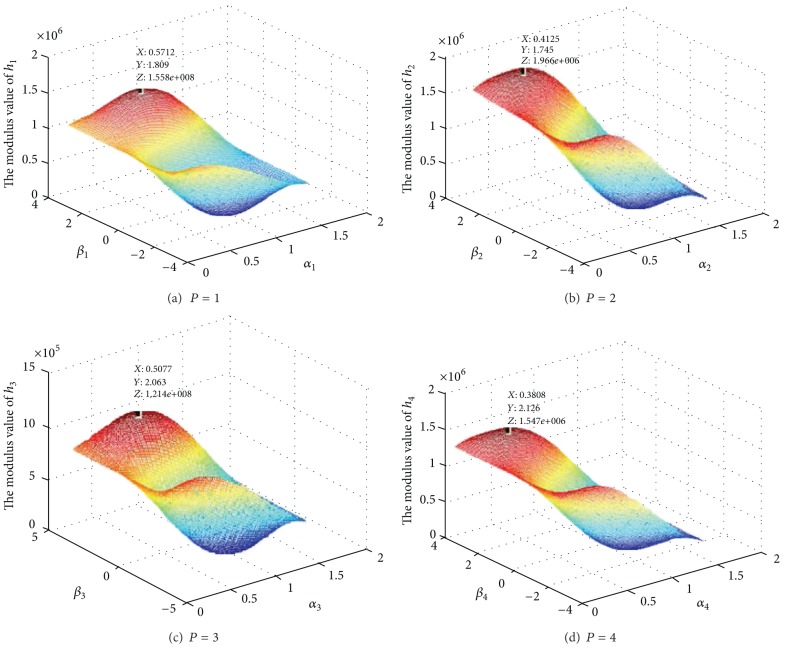
The distribution of modulus value of **h**.

Figure 5 depicts a three-dimensional distribution of modulus value of *h*_*p*_ when the orientation angle *α*_*p*_ and the ellipticity angle *β*_*p*_ of polarization ellipse are valued within the range: *α*_*p*_ ∈ [−*π*/2, *π*/2] and *β*_*p*_ ∈ [−*π*/4, *π*/4]. From Figure 3 we can see the proposed method. Comparing Tables 2 and 3 with Figure 5, we can prove that the proposed algorithm is reliable. 

**Figure 6:**
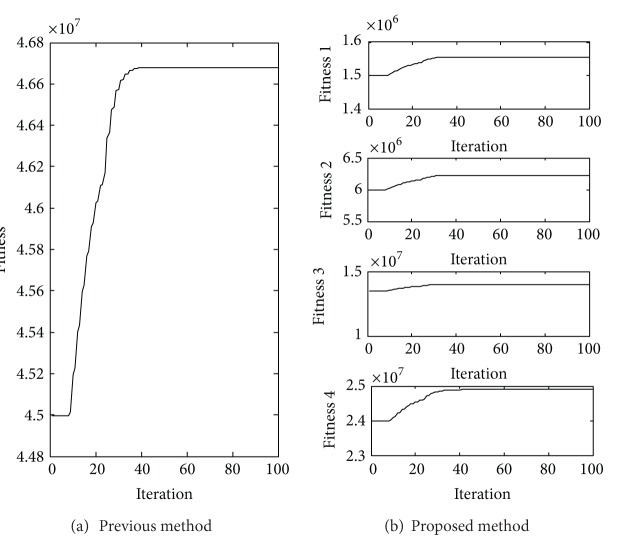
The fitness curves of two methods in special circumstance.

Here we select *P* = 4; *M* = 2; *μ* = 3; **s** = [2*i*, −1*i*, 0.5]^*T*^; **a**_1_ = [7 + 8*i*, 8 − 2*i*]^*T*^; **a**_2_ = 2**a**_1_; **a**_3_ = 3**a**_1_; **a**_4_ = 4**a**_1_ in special circumstance. From Figure 6, Table 4 and Table 5 we can see that the following experiment results get the same conclusion as Section 5 in detection performance analysis. However, from Table 6 we can see that the efficiency of the proposed method is 9 times more than the efficiency of the previous method. The numerical simulations are conducted to attest to the validity of the above theoretical analysis.

We consider two different cases (*q* = 3, *P* = 4, *μ* = 3) to illustrate the advantage of our optimization algorithm. In Case 1, the polarization state is fixed. In Case 2, the polarization state can be arbitrarily selected, and we use the proposed optimization algorithm in Case 2. When depicting the detection probability curves of Rao test and Wald test, the probability of false alarm is set to be 10^−4^, and we choose the statistical data model in [35].

**Figure 7:**
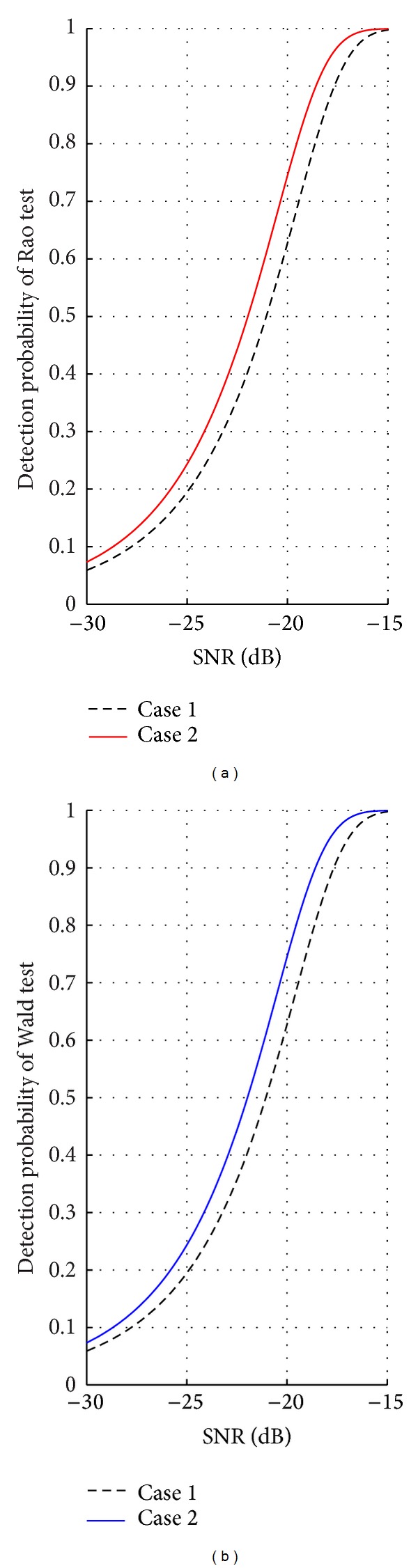
Performance comparisons of Rao test and Wald test between different cases.

We can see that the detection performance of both the detectors is indeed enhanced by utilizing the proposed algorithm. The gains with respect to the conventional case are approximately 1 dB in Case 2, respectively, when the detection probability is 0.9. Comparing Figures 7(a) and 7(b), we can find that the performances of Rao test and Wald test are exactly the same and it is attested to coincidence of Rao test and Wald test again.

## 7. Conclusions

In this paper, we study Rao test and Wald test for adaptive detection based on a DPA in partially homogeneous environment. Firstly, we derive the expressions for the probabilities of false alarm and detection of Rao test and Wald test with unknown noise covariance matrix structure based on a DPA, in the partially homogeneous environment. It is found from these derived expressions that Rao test and Wald test have the CFAR property. Secondly, the monotonicities of detection probability of the two detectors are proved, and a polarization optimization detection algorithm to enhance the detection performance is proposed. The improvement in the detection performance of the DPA is achieved by using the proposed algorithm to optimally select the polarization of the transmitted pulses. The theoretical analyses and the numerical simulations are conducted to attest to detection of the performance advantage of the proposed optimization algorithm. What is more, the proposed method was much more efficient than the previous method.

## Figures and Tables

**Table 1:** The maximum fitness function values got by two methods (×10^6^).

Methods	*P*	1	2	3	4
Proposed method	Fitness 1	1.5560	1.5560	1.5560	1.5560
	Fitness 2		1.9668	1.9668	1.9668
	Fitness 3			1.2143	1.2143
	Fitness 4				1.5471
	Sum	1.5560	3.5228	4.7371	6.2842

Previous method	Fitness	1.5560	3.5228	4.7371	6.2842

**Table 2:** The optimal polarization parameters got by two methods (rad).

(*α*_*p*_, *β*_*p*_)	Previous method	Proposed method
*α*_1_	0.5648	0.5648
*β*_1_	1.7805	1.7805
*α*_2_	0.4070	0.4070
*β*_2_	1.7996	1.7996
*α*_3_	0.5013	0.5013
*β*_3_	2.0538	2.0538
*α*_4_	0.3776	0.3776
*β*_4_	2.1467	2.1467

**Table 3:** The time cost by two methods (ms).

*P*	Previous method	Proposed method
1	47.139	43.702
2	164.46	87.175
3	354.16	130.52
4	457.63	178.48

**Table 4:** The maximum fitness function values got by two methods in a special circumstance (×10^7^).

Methods	*P*	4
Proposed method	Fitness 1	0.1556
	Fitness 2	0.6224
	Fitness 3	1.4004
	Fitness 4	2.4896
	Sum	4.6880

Previous method	Fitness	4.6880

**Table 5:** The optimal polarization parameters got by two methods in a special circumstance (rad).

(*α*_*p*_, *β*_*p*_)	Previous method	Proposed method
*α*_1_	0.5648	0.5648
*β*_1_	1.7805	1.7805
*α*_2_	0.5648	0.5648
*β*_2_	1.7805	1.7805
*α*_3_	0.5648	0.5648
*β*_3_	1.7805	1.7805
*α*_4_	0.5648	0.5648
*β*_4_	1.7805	1.7805

**Table 6:** The time cost by two methods in a special circumstance (ms).

Previous method	Proposed method
443.72	43.542
